# Supplementary material for: Conditional chemoconnectomics (cCCTomics) as a strategy for efficient and conditional targeting of chemical transmission
Source: eLife. 2024 Apr 30;12:RP91927. doi: 10.7554/eLife.91927 (PMC11060718; doi:10.7554/eLife.91927)
Supplement: Figure 3—source data 3. [file elife-91927-fig3-data3.zip › Figure 3 source data3 part 2.zip!/Figure 3-Source data 3-part2/1062-13-CRISPResso_Report_jt28fhr9.zip!/CRISPResso_on_1062_13/CRISPResso2_report.html]

CRISPResso2 Report


# CRISPResso2

### Analysis of genome editing outcomes from deep sequencing data

##### 1062\_13

##### CRISPResso2 run information

- Alignment statistics
- Run Parameters

Figure 1a: The number of reads in input fastqs, after preprocessing, and after alignment to amplicons.

Data: Mapping statistics

CRISPResso version: 2.2.12

Run completed: 2023-08-06 02:15:35

Amplicon sequence:

```
aacggacaaggagctaagggagcgtatacaacgagcacaggagcgcccatttcaaccgacagtgccagcccgtaaaaatgcaggaaatgagctacctacaggataattccaaggtggaggccctcaccaaggcggtgctcatatcgatactgggcgtggccatcgtcctgtccaacctcctcattatcgccacct
```

Guide sequence:

```
gcaggaaatgagctacctac
```

Command used:

```
CRISPResso -o CRISPRessoBatch_on_jt28fhr9 --name 1062_13 --min_paired_end_reads_overlap 10 --trimmomatic_command trimmomatic --needleman_wunsch_gap_open -20 --aln_seed_count 5 --needleman_wunsch_gap_extend -2 --amplicon_seq aacggacaaggagctaagggagcgtatacaacgagcacaggagcgcccatttcaaccgacagtgccagcccgtaaaaatgcaggaaatgagctacctacaggataattccaaggtggaggccctcaccaaggcggtgctcatatcgatactgggcgtggccatcgtcctgtccaacctcctcattatcgccacct --quantification_window_center -3 --needleman_wunsch_aln_matrix_loc EDNAFULL --prime_editing_pegRNA_extension_quantification_window_size 5 --amplicon_name gcaggaaatgagctacctac --n_processes 1 --write_cleaned_report --place_report_in_output_folder --max_rows_alleles_around_cut_to_plot 50 --needleman_wunsch_gap_incentive 1 --min_average_read_quality 0 --guide_seq gcaggaaatgagctacctac --aln_seed_len 10 --flash_command flash --prime_editing_pegRNA_scaffold_min_match_length 1 --min_single_bp_quality 0 --verbosity 3 --min_frequency_alleles_around_cut_to_plot 0.2 --min_bp_quality_or_N 0 --fastq_r1 CRISPResso_Input_Reads_d6e5af68-0914-47f6-a62d-bd62d01d2ee6.gz --max_paired_end_reads_overlap 100 --exclude_bp_from_left 15 --default_min_aln_score 50 --conversion_nuc_from C --exclude_bp_from_right 15 --conversion_nuc_to T --plot_window_size 20 --flexiguide_homology 80 --aln_seed_min 2 --quantification_window_size 20 --fastq_r2 CRISPResso_Input_Reads_e9bd2348-a8e2-40c2-ad34-a92efdbb5f58.gz
```

Parameters:

```
allele_plot_pcts_only_for_assigned_reference: False
aln_seed_count: 5
aln_seed_len: 10
aln_seed_min: 2
amplicon_min_alignment_score: 
amplicon_name: gcaggaaatgagctacctac
amplicon_seq: aacggacaaggagctaagggagcgtatacaacgagcacaggagcgcccatttcaaccgacagtgccagcccgtaaaaatgcaggaaatgagctacctacaggataattccaaggtggaggccctcaccaaggcggtgctcatatcgatactgggcgtggccatcgtcctgtccaacctcctcattatcgccacct
annotate_wildtype_allele: 
assign_ambiguous_alignments_to_first_reference: False
auto: False
bam_chr_loc: 
bam_input: 
bam_output: False
base_editor_output: False
bowtie2_index: 
coding_seq: 
conversion_nuc_from: C
conversion_nuc_to: T
crispresso1_mode: False
debug: False
default_min_aln_score: 50
discard_guide_positions_overhanging_amplicon_edge: False
discard_indel_reads: False
dsODN: 
dump: False
exclude_bp_from_left: 15
exclude_bp_from_right: 15
expand_allele_plots_by_quantification: False
expand_ambiguous_alignments: False
expected_hdr_amplicon_seq: 
fastq_output: False
fastq_r1: CRISPResso_Input_Reads_d6e5af68-0914-47f6-a62d-bd62d01d2ee6.gz
fastq_r2: CRISPResso_Input_Reads_e9bd2348-a8e2-40c2-ad34-a92efdbb5f58.gz
file_prefix: 
flash_command: flash
flexiguide_homology: 80
flexiguide_name: 
flexiguide_seq: None
force_merge_pairs: False
guide_name: 
guide_seq: gcaggaaatgagctacctac
ignore_deletions: False
ignore_insertions: False
ignore_substitutions: False
keep_intermediate: False
max_paired_end_reads_overlap: 100
max_rows_alleles_around_cut_to_plot: 50
min_average_read_quality: 0
min_bp_quality_or_N: 0
min_frequency_alleles_around_cut_to_plot: 0.2
min_paired_end_reads_overlap: 10
min_single_bp_quality: 0
n_processes: 1
name: 1062_13
needleman_wunsch_aln_matrix_loc: EDNAFULL
needleman_wunsch_gap_extend: -2
needleman_wunsch_gap_incentive: 1
needleman_wunsch_gap_open: -20
no_rerun: False
output_folder: CRISPRessoBatch_on_jt28fhr9
place_report_in_output_folder: True
plot_histogram_outliers: False
plot_window_size: 20
prime_editing_nicking_guide_seq: 
prime_editing_override_prime_edited_ref_seq: 
prime_editing_override_sequence_checks: False
prime_editing_pegRNA_extension_quantification_window_size: 5
prime_editing_pegRNA_extension_seq: 
prime_editing_pegRNA_scaffold_min_match_length: 1
prime_editing_pegRNA_scaffold_seq: 
prime_editing_pegRNA_spacer_seq: 
quantification_window_center: -3
quantification_window_coordinates: None
quantification_window_size: 20
save_also_png: False
split_interleaved_input: False
stringent_flash_merging: False
suppress_plots: False
suppress_report: False
trim_sequences: False
trimmomatic_command: trimmomatic
trimmomatic_options_string: 
use_legacy_insertion_quantification: False
verbosity: 3
write_cleaned_report: True
write_detailed_allele_table: False
zip_output: False
```

Running log

##### Allele assignments

- Piechart
- Barplot

Figure 1b: Alignment and editing frequency of reads as determined by the percentage and number of sequence reads showing unmodified and modified alleles.

Data: Quantification of editing

Figure 1c: Alignment and editing frequency of reads as determined by the percentage and number of sequence reads showing unmodified and modified alleles.

Data: Quantification of editing

##### Nucleotide composition

Hover your mouse over the bottom image to zoom in on a specific region.  
Figure 2a: Nucleotide distribution across amplicon. At each base in the reference amplicon, the percentage of each base as observed in sequencing reads is shown (A = green; C = orange; G = yellow; T = purple). Black bars show the percentage of reads for which that base was deleted. Brown bars between bases show the percentage of reads having an insertion at that position.

Data: Nucleotide frequency table

Figure 2b: Nucleotide distribution around the sgRNA gcaggaaatgagctacctac.

Data: Nucleotide frequency in quantification window

##### Modification lengths

- Summary
- Indels

Figure 3a: Frequency distribution of alleles with indels (blue) and without indels (red). Note that histograms are clipped to show 99% of the data. To show all data, run using the parameter '--plot\_histogram\_outliers'. (Minimum -56 not shown)

Data: Indel histogram

Figure 3b: Left panel, frequency distribution of sequence modifications that increase read length with respect to the reference amplicon, classified as insertions (positive indel size). Middle panel, frequency distribution of sequence modifications that reduce read length with respect to the reference amplicon, classified as deletions (negative indel size). Right panel, frequency distribution of sequence modifications that do not alter read length with respect to the reference amplicon, which are classified as substitutions (number of substituted positions shown).

Data: Insertions frequency

Data: Deletions Frequency

Data: Substitutions Frequency

##### Indel characterization

- All Modifications Combined
- All Modifications by Type
- Modifications in Quantification Window
- Indel Lengths

Figure 4a: Combined frequency of any modification across the amplicon. Modifications outside of the quantification window are also shown.

Figure 4b: Frequency of insertions (red), deletions (purple), and substitutions (green) across the entire amplicon, including modifications outside of the quantification window.

Data: Modification frequency

Figure 4c: Frequency of insertions (red), deletions (purple), and substitutions (green) across the entire amplicon, considering only modifications that overlap with the quantification window.

Data: Modification frequency in quantification window

Figure 4d: Position dependent insertion size(left) and deletion size (right), including only modifications that overlap with the quantification window.

##### Allele plots

Figure 9: Visualization of the distribution of identified alleles around the cleavage site for the sgRNA gcaggaaatgagctacctac. Nucleotides are indicated by unique colors (A = green; C = red; G = yellow; T = purple). Substitutions are shown in bold font. Red rectangles highlight inserted sequences. Horizontal dashed lines indicate deleted sequences. The vertical dashed line indicates the predicted cleavage site.

Data: Allele frequency table

If you like CRISPResso2 please support us by citing it in your work:   
Clement K, Rees H, Canver MC, Gehrke JM, Farouni R, Hsu JY, Cole MA, Liu DR, Joung JK, Bauer DE, Pinello L.  
CRISPResso2 provides accurate and rapid genome editing sequence analysis.   
Nat Biotechnol. 2019 Mar; 37(3):224-226. doi: 10.1038/s41587-019-0032-3. PubMed PMID: 30809026.

© Copyright Kendell Clement and Luca Pinello

Tweet about CRISPresso2!
